# Machine Learning-Based Predictive Modeling of Postpartum Depression

**DOI:** 10.3390/jcm9092899

**Published:** 2020-09-08

**Authors:** Dayeon Shin, Kyung Ju Lee, Temidayo Adeluwa, Junguk Hur

**Affiliations:** 1 Department of Food and Nutrition, Inha University, Incheon 22212, Korea; dyshin@inha.ac.kr; 2 Department of Obstetrics and Gynecology, Korea University Medical Center, Seoul 02841, Korea; drlkj52551@korea.ac.kr; 3 Department of Biomedical Sciences, University of North Dakota, Grand Forks, ND 58202, USA; temidayo.adeluwa@und.edu

**Keywords:** postpartum depression, machine learning, predictive modeling, Pregnancy Risk Assessment Monitoring System (PRAMS)

## Abstract

Postpartum depression is a serious health issue beyond the mental health problems that affect mothers after childbirth. There are no predictive tools available to screen postpartum depression that also allow early interventions. We aimed to develop predictive models for postpartum depression using machine learning (ML) approaches. We performed a retrospective cohort study using data from the Pregnancy Risk Assessment Monitoring System 2012–2013 with 28,755 records (3339 postpartum depression and 25,416 normal cases). The imbalance between the two groups was addressed by a balanced resampling using both random down-sampling and the synthetic minority over-sampling technique. Nine different ML algorithms, including random forest (RF), stochastic gradient boosting, support vector machines (SVM), recursive partitioning and regression trees, naïve Bayes, k-nearest neighbor (kNN), logistic regression, and neural network, were employed with 10-fold cross-validation to evaluate the models. The overall classification accuracies of the nine models ranged from 0.650 (kNN) to 0.791 (RF). The RF method achieved the highest area under the receiver-operating-characteristic curve (AUC) value of 0.884, followed by SVM, which achieved the second-best performance with an AUC value of 0.864. Predictive modeling developed using ML-approaches may thus be used as a prediction (screening) tool for postpartum depression in future studies.

## 1. Introduction

Postpartum depression is a mood disorder that affects up to 15% and 13% of mothers after childbirth in the United States and worldwide, respectively [1,2]. Postpartum depression is known to be associated with adverse maternal, child, and infant outcomes, such as low breastfeeding initiation, short duration and decreased levels of breastfeeding self-efficacy [3], poor maternal and infant bonding [4,5], and impaired mental and motor development in the infant [6]. Previous research has revealed that risk factors for postpartum depression include a history of mental illness, such as past history of postpartum depression, other depression or psychiatric illnesses, and a family history of affective disorder [7]; low social support [8]; poor marital relationship [9]; pregnancy-related complications, including emergency cesarean sections [10]; unplanned/unwanted pregnancy [11]; stressful life events during pregnancy [12]; and preterm birth [13]. Although these independent risk factors for postpartum depression are known, little is known about the predictive modeling of postpartum depression that includes maternal and paternal risk factors. One of the objectives of the Healthy People 2020 initiative is to decrease the proportion of women delivering live births who experience postpartum depressive symptoms, so it is imperative to develop a screening tool for postpartum depression for prevention and intervention purposes.

Machine learning (ML) methods provide advantages for the prediction of various diseases. Specifically, ML has been applied for predictive models of various health outcomes, such as metabolic syndrome [14], cerebral infarction [15], heart failure [16], and Alzheimer’s disease [17]. In line with such diseases, predictive models for postpartum depression in adolescent and adult mothers have been developed that include information such as maternal race, pregnancy intention, socioeconomic status, prior depression, mental health during pregnancy, stressors, and social support by overlaying receiver operating characteristic (ROC) plots and through comparisons of the c-statistics [18] using data from the Rhode Island Pregnancy Risk Assessment Monitoring System (PRAMS). However, to the best of our knowledge, there are no predictive tools available to screen postpartum depression that also allow early interventions based on diverse ML approaches. The overall study objective was to develop and validate ML-based predictive models for postpartum depression using both maternal and paternal characteristics from the PRAMS 2012–2013 data.

## 2. Materials and Methods

### 2.1. Study Participants

**Figure 1:**
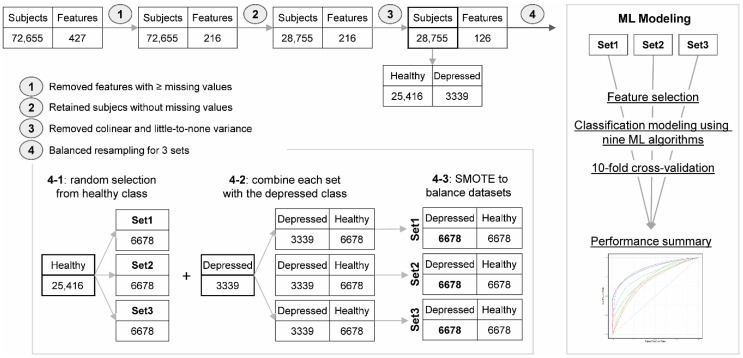
The overall process of data preprocessing and imbalance handling. Data preprocessing included (1) removal of features with more than 10,000 missing values; (2) removal of observations with missing values; and (3) removal of non-informative features with colinear or little-to-none variance. The resulting “cleansed” dataset was split to create three distinct datasets and synthetic minority oversampling technique (SMOTE) was used to balance these datasets in (4), which were used for classification model building and evaluation.

We obtained the complete PRAMS 2012–2013 data from the Centers for Disease Control and Prevention (CDC). PRAMS collects state-specific, population-based data on maternal characteristics and experience before, during, and after pregnancy in the United States. A PRAMS sample of women who recently delivered live births was selected from the state birth certificate registries, and these women were asked to participate in the PRAMS survey [19]. Each participating state drew a stratified systematic sample of 100 to 250 new mothers every month from selected eligible birth certificates [19]. Most states oversample low-weight births, and each participating state generally samples between 1300 and 3400 women per year [19]. Sampling fractions in PRAMS range from 1 in 1 (for very low birth weight strata in small states) to approximately 1 in 211 (for normal birth weight and nonminority strata in populous states) [19]. The PRAMS 2012–2013 data included a total of 72,540 participants, and we selected 28,755 records for this study after removing missing or unknown information and cleansing the data (Figure 1).

### 2.2. Target Variable for Predictive Modeling: Postpartum Depression

The diagnosis of postpartum depression was based on a modified version of the Patient Health Questionnaire-2 (PHQ-2). Women were asked two questions: “Since your new baby was born, how often have you felt down, depressed, or hopeless?” and “Since your new baby was born, how often have you had little interest or pleasure in doing things?” Women responding with “always” or “often” to one or both of these questions were deemed to be “postpartum depressive”. This approach was previously evaluated and achieved a sensitivity of 63% and specificity of 83% for identifying postpartum depression cases [20].

### 2.3. Machine Learning Methods for Predictive Modeling

For our classification modeling of postpartum depression classification, we used the statistical programming language, R (Version 4.0.0), and the Classification And Regression Training (caret) package [21].

#### 2.3.1. Resampling to Address Group Imbalance

The PRAMS data are imbalanced with the healthy class comprising most of the data (*n* = 25,416; 88%). This imbalance persisted even after cleansing the data set. In the ML-based classification approach, imbalanced data may lead to a significantly poor classification accuracy [22]. To address the imbalanced data issue, we randomly selected observations from the larger, healthy class, three times such that each selection is unique (Figure 1). Thereafter, each unique selection was combined with the smaller, postpartum depression group to generate three unique combined datasets (named “Set 1”, “Set 2”, and “Set 3”). We treated each dataset independently and used the synthetic minority oversampling technique (SMOTE) [23], which is a widely used oversampling method to balance the data imbalance issue. We randomly selected twice the size of the depressed class (3339 × 2, or 6678 observations). Instead of replicating the existing members in the minority group, SMOTE creates synthetic members based on nearest neighbors judged by Euclidean distances between the data points in the feature space.

#### 2.3.2. Feature Selection (Inputs for Predictive Modeling: Maternal and Paternal Factors)

Feature selection is the process of reducing the number of variables in a predictive model to reduce the computational cost of modeling and to improve the performance. We systematically evaluated five data-driven feature selection methods, including recursive feature elimination (RFE) [24], information gain [25], Relief [26], stepwise generalized linear modeling (glmStepAIC) [27], and a bagging-based selection-by-filter (SBF) method [21]. The overall classification performance of five RF models on Set 1 with selected features by five methods, as well as a reduction in features and computation time were considered to select the most appropriate feature selection method for our current study.

#### 2.3.3. Classification Modeling

Nine ML algorithms from the R caret package [21], including k-nearest neighbor (kNN), recursive partitioning (RPART; a decision tree-based method), support vector machine (SVM), stochastic gradient boosting (GBM), random forest (RF), neural network (NNET), naïve Bayes (NB), logistic regression (LR), and AdaBoost, were used in the current study. To evaluate the classification models, a 10-fold cross-validation strategy was used, where the original samples were randomly partitioned into 10 equal-sized subsamples and a single subsample was retained as validation data for testing the model built using the other nine subsamples. We ran these algorithms on all three independent datasets (Sets 1, 2, and 3).

The area under curve (AUC) was used as the primary performance metrics in the current study. AUC is a widely used metric for binary classification problems and describes the ability of the model to separate the classes into healthy or depressed classes. Other metrics include (1) sensitivity, also known as the true positive rate or recall, which describes what proportion of the correctly classified depressed cases out of all depressed cases. Essentially, sensitivity describes the probability that the model predicts a case as “depressed”, given that the patient is actually depressed; (2) specificity, also known as the true negative rate, is the proportion of the correctly classified healthy cases by the model out of all healthy classes from the dataset; (3) accuracy takes into consideration both the sensitivity and specificity of the model and describes what proportion of all cases or subjects were correctly classified by that model. These three metrics are of clinical importance in this study. Precision focuses on the positive class, in the postpartum depression class in this study, and it describes the proportion correctly predicted cases out of all cases labelled as depressed by the model; and F1 score, which is a weighted average of precision and recall (sensitivity). All these metrics range from 0 to 1 with 0 representing a poor metric and 1 depicting a perfect metric. The closer the metrics are to 1, the better the models are.

### 2.4. Statistical Analyses

The frequency and distribution of maternal characteristics by the status of postpartum depression were assessed by cross-tabulation with Chi-squared statistics. Logistic regression models were used to assess the relationships between maternal characteristics as independent variables and postpartum depression as the outcome. All analyses were performed using SAS version 9.4 survey procedures (SAS Institute, Cary, NC, USA) after applying a weighted complex sampling design.

### 2.5. Ethical Approval

Ethical review from an institutional review board approval was not required because PRAMS was a publicly available dataset that contained no personally identifiable information.

## 3. Results

### 3.1. Maternal Demographics and Lifestyle Factors

Maternal demographic factors are presented by the status of postpartum depression. The status of postpartum depression significantly differed by maternal age, maternal race/ethnicities, education, small-for-gestational-age based on the 10th percentile, pre-pregnancy exercise for more than three days, depression before pregnancy, drinking three months before pregnancy, changing smoking in the last three months of pregnancy and postpartum period, and marital status (all *p*-values < 0.05). Women without postpartum depression were more likely to have greater education (42.6%). They were more likely to be nonsmokers (86.5%), married (70%), and did not have depression before pregnancy (92.2%). Women with postpartum depression were more likely to have less education (42.3%) and had depression before pregnancy (23.7%) (Table 1).

### 3.2. Association of Maternal Demographics and Lifestyle Factors with Postpartum Depression

Mothers aged ≤19 years had greater odds of experiencing postpartum depression compared with mothers aged 20 to 29 years (OR 1.50, 95% CI 1.07–2.09). Mothers who had an education of 0–12 years and 13–15 years had increased odds for postpartum depression compared with those with more than 16 years of education (OR 1.59, 95% CI 1.27–2.00; OR 1.45, 95% CI 1.19–1.77, respectively). Mothers who delivered small-for-gestational-age infants had greater odds of having postpartum depression (OR 1.37, 95% CI 1.11–1.69). Prior pregnancy depression was associated with increased odds of postpartum depression (OR 3.15, 95% CI 2.60–3.80). Mothers who drank alcohol three months before pregnancy had lower odds of having postpartum depression (OR 0.84, 95% CI 0.72–0.99). In the meantime, mothers with the number of cigarettes reduced had higher odds of having postpartum depression (OR 2.58, 95% CI 1.06–6.29, respectively). Mothers with other marital statuses compared with married mothers had increased odds of postpartum depression (OR 1.52, 95% CI 1.27–1.83) (Table 2).

### 3.3. Prediction Modeling

The initial PRAMS 2012–2013 dataset included a total of 72,540 records. All of these records have at least one missing value in them, necessitating the proper cleansing of the dataset. Our approach to cleansing this dataset included removing features with at least 10,000 missing values, before selecting for complete records. We also filtered out collinear features and employed several traditional cleansing steps before model building as illustrated in Figure 1. The final “cleansed” dataset included 28,755 valid records with 25,416 healthy and 3339 depressed cases. We split this cleansed dataset into three unique sets and used SMOTE to improve the ratio of normal to postpartum depression cases to 1.

#### 3.3.1. Feature Selection for Modeling

We evaluated five different data-driven feature selection methods using RF modeling on Set 1 and their resulting classification performance is summarized in Table S1. All five methods achieved comparable and high AUC values (0.871–0.885). We selected Relief algorithm as our method of feature selection, which achieved an AUC value of 0.885, showed a substantial reduction in the number of features (from 126 to 99), and reduced in computational time.

The selected features by Relief included maternal age, race/ethnicity, education, marital status, pre-pregnancy body mass index (BMI), smoking status, drinking status, previous history of depression, physical activity, number of previous live births, gender of the infant, stress-related features, multivitamin use, small-for-gestational-age, large-for-gestational-age, and the Kotelchuck index for the responder (a clinical metric describing the adequacy of prenatal care). These factors were previously reported to be linked to postpartum depression [9,18,28,29,30,31].

#### 3.3.2. Performance Evaluation of Classification Models

Classification modeling was performed to predict the binary class of postpartum depression (healthy subjects and depressive subjects) using features returned by a multivariate feature selection method, Relief. A total of 99, 86, and 95 features were selected by Relief on Set 1, Set 2, and Set 3, respectively, with 47 features common to all three sets.

**Figure 2:**
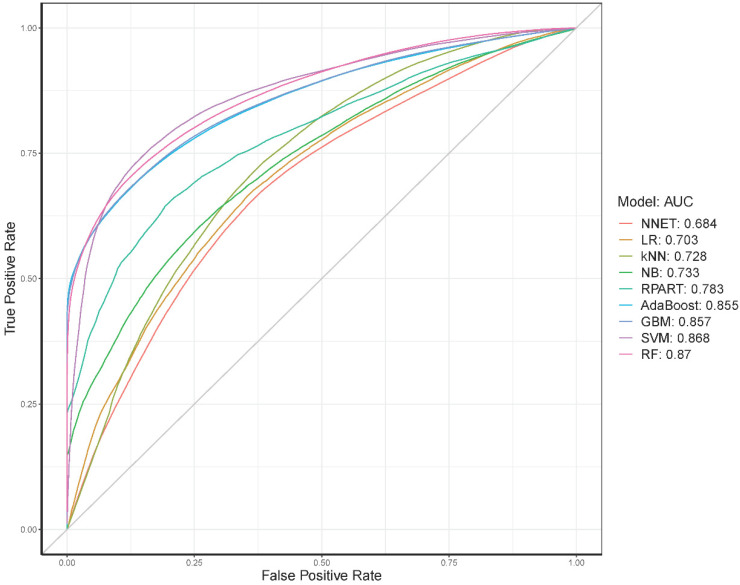
Receiver operating characteristic (ROC) curves for the nine machine learning (ML) models in Set 1 in predicting postpartum depression. Ten-fold cross-validation was used to build and evaluate the prediction models. Different colors represent the top four machine learning classifiers used in this study. The false positive rate is equal to (1–specificity). The gray line is the reference corresponding to the performance of a classifier that completely and randomly classifies the condition.

The classification performance of the nine ML models on Set 1 is illustrated in a ROC curve (Figure 2). Table 3 summarizes the performance average across all three datasets, while the individual performance on each of the three sets is given in Tables S2–S4. Overall, the RF method achieved the highest area under the ROC curve (AUC) value, 0.884, followed by SVM with an AUC of 0.864. All classifiers achieved better classification accuracy than a random model (the gray diagonal line indicating AUC = 0.500 in Figure 2).

The average AUC across three datasets ranged from 0.704 (NNET) to 0.884 (RF). These results imply little variation across our models and that our models do not overfit the data—a characteristic of good ML models.

### 3.4. Important Features Ranked by Each ML Algorithm

Features contribute differently to each model; we used the varImp function of caret package to calculate variable importance in each model. The top 20 most contributing features from the four best-performing models (RF, SVM, GBM, and AdaBoost) were combined and ranked based on their inclusion in these four models. In total, these models returned 50 top twenty features (Table S5), nine of which were within the top 20 in at least three models, given in Table 4 with their rankings in each model. The most frequent and important features include exposure to stress during pregnancy, having depression before pregnancy, weeks spent breastfeeding the baby, income, maternal education, maternal education, dental hygiene before pregnancy, and the gender of the baby.

## 4. Discussion

In the present study, significant risk factors for postpartum depression included maternal age, education, marital status, small-for-gestational-age based on 10th percentile, depression before pregnancy, and smoking behavior change from the last three months of pregnancy to postpartum period. Mothers aged ≤ 19 years had increased odds of having postpartum depression, as did mothers with education of 0–12 years and 13–15 years and small-for-gestational-age infants. Those with depression before pregnancy, those who reduced cigarette smoking from the last three months of pregnancy to postpartum period and those who were unmarried had increased odds of having postpartum depression. In contrast to our finding, for adult mothers over the age of 25 had increased odds of having postpartum depressive symptoms [18].

In this study, prenatal depression was associated with postpartum depressive symptoms; the high prevalence of depression and suicidal ideation during adolescence and young adulthood may reflect family societal pressure on women to achieve high academic standards and perform traditional gender roles [32,33]. The preference for a male infant is one of the significant determinants for postpartum depression in Indian [34] and Chinese women [35,36]. The increased risk for postpartum depression among women with female infants could be explained by poor postnatal support from family members, especially husbands and parents [37]. In our study, top features returned by our machine learning-based models included exposure to stress during pregnancy, having depression before pregnancy, weeks spent breastfeeding the baby, income, dental hygiene before pregnancy, and the gender of the baby. Life stress and a history of depression and have been the most significant predictors for postpartum depression [38]. In particular, exposure to stress changes the levels of hormones in the hypothalamus-pituitary-adrenal (HPA) axis, especially cortisol level, and depressed individuals demonstrate abnormal HPA axis function by releasing high levels of cortisol [39,40]. Also, women’s oral health may influence many pregnancy outcomes beyond postpartum depression. Maternal periodontal disease has been linked to low birth weight and preterm birth [41,42].

Even though the present study did not explore the gender of the infant, this previous finding may be relevant to our study, in that Asian mothers may have displayed a high prevalence of postpartum depression with a female infant. In our study, unmarried mothers had increased odds of having postpartum depression (OR 1.26, 95% CI 1.12–1.43). This finding is consistent with a previous report, where unmarried women had significant odds of having postpartum depressive symptoms in the Rhode Island-specific PRAMS 2004–2008 [18]. This study suggests that marital status is associated with experiencing postpartum depression, which is consistent with our study findings.

Regarding ML classifiers, our study found that RF achieved the best performances for predicting postpartum depression, with a classification accuracy value of 0.791 and an AUC value of 0.884, respectively. Similar to our study findings, one study using data from the Rhode Island PRAMS [18] developed a forward selection-based predictive model for postpartum depression, which achieved an AUC value of 0.79. The risk factors included pregnancy intention, race, stress, economic status, and social support. Tortajada et al. developed another prediction model for postpartum depression using multilayer perceptrons and pruning for pregnant Spanish women using data from seven Spanish general hospitals from 2003–2004 [43]. Their approach of using multilayer perceptrons showed good performance for prediction of postpartum depression, where the best model (the subject model with no pruning) achieved a sensitivity of 0.84, a specificity of 0.81, and an AUC value of 0.82. Using 45 Iranian depressive patients and 45 normal subjects, Hosseinifard et al. [44] employed logistic regression classifiers that achieved the highest classification accuracy of 83.3%. Combining multiple algorithms, including linear discriminant analysis (LDA), logistic regression (LR), and kNN, the accuracy of classification was improved by 6.7%, reaching an overall accuracy of 90%. Jimenez-Serrano et al. [45] employed NB, logistic regression, SVM, and artificial neural network (ANN) methods, where NB achieved the best balance between sensitivity and specificity. In their modeling, logistic regression achieved the highest AUC value of 0.77. Compared with these previous modeling studies, our ML models demonstrated comparable or better overall prediction performance. It is worthy of note that the best performing algorithms in this study are known to have implicit feature selection processes and will usually select their own best set of predictive features.

Our study demonstrates several strengths. First, PRAMS collects state-specific, population-based data on maternal attitudes and experiences before, during, and shortly after pregnancy in a standardized data collection methodology [46] and covers 83% of all U.S. births [47]. Furthermore, a number of significant features were selected in a data-driven approach to building the ML-based prediction models.

Despite these strengths, there are also a few limitations. Postpartum depression was based on mothers’ self-reports, rather than a medical diagnosis; therefore, there may be information bias. There was a lot of missing data on sociodemographic and lifestyle variables in this study (*n* = 28,755 vs. 72,540), and it is possible that the results in the non-response or missing population could differ from those of the response population. PHQ-2 was used to diagnose postpartum depression. PHQ-2 is a screening tool that measures the presence of symptoms consistent with major depression but does not indicate the etiologies of postpartum depression although knowing the etiologies of postpartum depression is significant for a comprehensive diagnostic process of postpartum depression [48]. PHQ-2 includes two items from the PHQ-9 regarding the frequency of depressed mood and anhedonia over the past two weeks as a first-step approach, and it is not intended to reveal the severity of depression nor used as the final diagnosis of depression. Patients who screen positive from the PHQ-2 should be further evaluated with the PHQ-9 to determine whether they are diagnosed with a depressive disorder [49].

We used the SMOTE oversampling approach to address the strong imbalance between the healthy and depression group. To ensure that we do not overfit the models, we used a cross-validation approach to model building. We also randomly divided the available dataset into three distinct datasets and treated each one independent of the other. Nevertheless, further evaluation of the models using an independent cohort would be needed. While our current approach employs traditional machine learning methods, an application of advanced artificial neural network architecture integrating electronic health records needs to be explored in the future [50]. We will employ ensemble methods, combining the outcomes of multiple ML methods into one, to improve the prediction of our models. We will also further reduce the number of features to the most relevant ones while keeping the high prediction performance and will evaluate simple-to-use nomograms based on our models for clinical use.

Additionally, we observed that some well-known features suggested by the literature were excluded from our final models. These features have been reported by previous literature [9,18,28,29,30,31] to be highly-correlated with depression but automatically removed in our study because they had a lot of missing values in the original data. For example, women whose babies were dead at the time of responding to the survey were seven times more likely to develop postpartum depression (Table S6). Unfortunately, the feature corresponding to this response was removed due to our data-cleansing approach. Future directions in this study will involve making extensive use of these sparse features that have a high correlation with the development of postpartum depression. Lastly, even though we used maternal inputs for predictive modeling since postpartum depression varies across racial and ethnic groups, we could not consider cultural variations in the experiences and expression of emotional distress that may lead to the under detection of misidentification of postpartum depression [51].

## 5. Conclusions

We used nine ML algorithms to build predictive models for postpartum depression. RF, AdaBoost, GBM, and SVM, in general, achieved the highest performance in predicting postpartum depression. ML-based predictive modeling using features including maternal age, race/ethnicity, education, number of previous live births, small-for-gestational-age based on the 10th percentile, various stress-related factors, pre-pregnancy exercise for more than three days, depression before pregnancy, drinking for three months before pregnancy, smoking behavior change from the last three months of pregnancy to postpartum period, maternal pre-pregnancy BMI, and other related features. This tool may thus be used as a prediction (screening) tool for postpartum depression in future studies.

## Figures and Tables

**Table 1:** Maternal demographic by the status of postpartum depression.

	No Postpartum Depression (*n* = 25,416)		Postpartum Depression (*n* = 3339)		
	*n*	Wt’d %	*n*	Wt’d %	*p*-Value
Maternal Age (years)					
≤19	1332	3.8	354	9.2	<0.0001
20–29	13,012	50.7	1867	56.7	
30–39	10,341	42.6	1045	31.7	
≥40	731	2.9	73	2.4	
Maternal Race/Ethnicity					
American Indian or Alaskan Native	897	1.0	156	1.9	<0.0001
Asian	1743	5.0	234	5.6	
Black	2834	9.4	570	13.4	
Hawaiian	396	0.4	36	0.3	
White or other non-white	18,487	81.9	2170	75.9	
Mixed race	1059	2.4	173	3.0	
Maternal Education					
0–12 years	8059	28.1	1559	42.3	<0.0001
13–15 years	7654	29.3	1057	32.6	
≥16 years	9703	42.6	723	25.1	
Marital Status					
Married	16,843	70.0	1613	51.2	<0.0001
Other	8573	30.0	1726	48.8	
Number of Previous Live Births					
0	11,106	42.6	1410	43.1	0.3415
1	7946	33.0	992	31.2	
≥2	6364	24.3	937	25.7	
Small for Gestational Age Based on 10th Percentile					
Yes	3829	8.7	628	12.4	<0.0001
No	21,587	91.3	2711	87.6	
Pre-pregnancy Exercise 3+ Days					
No	12,504	49.0	1892	55.3	<0.0001
Yes	12,912	51.0	1447	44.7	
Depression Before Pregnancy					
No	23,227	92.2	2474	76.3	<0.0001
Yes	2189	7.8	865	23.7	
Drinking 3 Months Before Pregnancy					
No	10,157	36.6	1452	41.0	0.0018
Yes	15,259	63.4	1887	59.0	
Changing Smoking Last 3 Months of Pregnancy & Postpartum Period					
Nonsmoker	21,588	86.5	2377	75.4	<0.0001
Smoker who quit	229	0.7	46	1.1	
Number of cigarettes reduced	110	0.4	43	1.6	
Number of cigarettes same/more	2271	7.7	593	14.1	
Nonsmoker resumed	1218	4.7	280	7.9	
Maternal Pre-pregnancy BMI (kg/m^2^)					
Underweight (≤18.5)	1044	3.5	200	5.4	<0.0001
Normal (18.5–25)	12,648	51.9	1440	45.4	
Overweight (25–30)	6131	24.0	823	24.8	
Obese (≥30)	5593	20.6	876	24.3	

Wt’d %: Weighted percentage. *p*-value was calculated by Chi-square tests.

**Table 2:** Associations between maternal demographics and lifestyle factors and postpartum depression.

	OR	(95% CI)
Maternal Age (years)		
≤19	1.50 *	(1.07–2.09)
20–29	1.00	
30–39	0.91	(0.77–1.07)
≥40	0.96	(0.62–1.50)
Maternal Race/Ethnicity		
American Indian or Alaskan Native	1.53	(0.93–2.50)
Asian	1.26	(0.78–2.02)
Black	1.24	(0.82–1.87)
Hawaiian	1.03	(0.16–6.76)
White or other non-white	1.00	
Mixed race	1.30	(0.87–1.93)
Maternal Education		
0–12 years	1.59 *	(1.27–2.00)
13–15 years	1.45 *	(1.19–1.77)
≥16 years	1.00	
Marital Status		
Married	1.00	
Other	1.52 *	(1.27–1.83)
Number of Previous Live Births		
0	1.00	
1	0.95	(0.80–1.14)
≥2	1.05	(0.86–1.29)
Small for Gestational Age Based on 10th Percentile		
Yes	1.37 *	(1.11–1.69)
No	1.00	
Pre-pregnancy Exercise 3+ Days		
No	1.00	
Yes	0.97	(0.84–1.13)
Depression Before Pregnancy		
No	1.00	
Yes	3.15 *	(2.60–3.80)
Drinking 3 Months Before Pregnancy		
No	1.00	
Yes	0.84 *	(0.72–0.99)
Changing Smoking Last 3 Months of Pregnancy & Postpartum Period		
Nonsmoker	1.00	
Smoker who quit	1.29	(0.62–2.66)
Number of cigarettes reduced	2.58 *	(1.06–6.29)
Number of cigarettes same/more	1.12	(0.87–1.44)
Nonsmoker resumed	1.19	(0.86–1.63)
Maternal Pre-pregnancy BMI (kg/m^2^)		
Underweight (≤18.5)	1.22	(0.86–1.74)
Normal (18.5–25)	1.00	
Overweight (25–30)	1.16	(0.97–1.38)
Obese (≥30)	1.20	(0.99–1.45)

* *p*-value < 0.05. OR: odds ratios, 95% CI: 95% confidence intervals.

**Table 3:** Average metrics of nine ML models across all independent datasets.

Model	AUC	Sensitivity	Specificity	Accuracy	Precision	F1
RF	0.884	0.732	0.865	0.791	0.839	0.776
SVM	0.864	0.791	0.788	0.789	0.789	0.789
GBM	0.859	0.695	0.868	0.781	0.839	0.760
AdaBoost	0.857	0.722	0.835	0.778	0.813	0.765
NB	0.793	0.578	0.853	0.675	0.709	0.647
RPART	0.789	0.658	0.807	0.731	0.772	0.708
kNN	0.776	0.925	0.455	0.641	0.593	0.715
LR	0.707	0.628	0.683	0.655	0.665	0.646
NNET	0.704	0.650	0.660	0.650	0.649	0.651

AUC: area under the curve.

**Table 4:** The most contributing features belonging to at least three of the best four models.

Features	Frequency	RF Rank	GBM Rank	AdaBoost Rank	SVM Rank	Description
BF5WEEKS	4	2	2	5	3	Number of weeks spent breastfeeding the baby
BPG_DEPRS	4	3	3	4	7	Depression before pregnancy
MAT_AGE_NAPHSIS	4	9	5	13	9	Maternal age grouped
STRS_T_G	4	1	1	1	1	Total number of stresses during the 12 months before childbirth grouped
INCOME7	3	6	15	NA	2	Total household income during the 12 months before childbirth
MAT_ED	3	7	8	NA	4	Maternal education
PRE_DEPR	3	NA	12	19	16	Pre-pregnancy check for depression/anxiety
PREG_TRY	3	NA	6	6	17	Trying to get pregnant
STRS_BIL	3	12	7	NA	5	Stress—couldn’t pay rent, mortgage, or other bills

NA: This feature was not ranked in the top 20 features for that model; STRS: stress; labels are from the PRAMS codebook.

## Supplementary Materials

The following are available online at https://www.mdpi.com/2077-0383/9/9/2899/s1, Table S1: RF-based performance evaluation of five data-driven feature selection methods, Table S2: classification performance of nine ML algorithms in predicting postpartum depression on Set 1, Table S3: classification performance of nine ML algorithms in predicting postpartum depression on Set 2, Table S4: classification performance of nine ML algorithms in predicting postpartum depression on Set 3, Table S5: top 20 contributing features in the four best performing models, Table S6: survey question: infant living at the time of the PRAMS report.
