# Supplementary material for: Machine Learning-Based Predictive Modeling of Postpartum Depression
Source: J Clin Med. 2020 Sep 8;9(9):2899. doi: 10.3390/jcm9092899 (PMC7564708; doi:10.3390/jcm9092899)
Supplement: Supplementary file 1 [file jcm-09-02899-s001.pdf]

Journal of
Clinical Medicine

Article

Machine learning-based predictive modeling of postpartum depression

Supplementary Materials

Dayeon Shin [1,$], Kyung Ju Lee [2,$], Temidayo Adeluwa [3,$], and Junguk Hur [3,*]

[1] Department of Food and Nutrition, Inha University, Incheon, 22212, Republic of Korea; dyshin@inha.ac.kr
[2] Department of Obstetrics and Gynecology, Korea University Medical Center, Seoul, 02841, Republic of Korea; drlkj52551@korea.ac.kr
[3] Department of Biomedical Sciences, University of North Dakota, Grand Forks, North Dakota, 58202, USA; temidayo.adeluwa@und.edu; junguk.hur@med.und.edu
$ These authors contributed equally to the work.
* Correspondence: junguk.hur@med.und.edu; Tel.: +1-701-777-6814

Table S1. RF-based performance evaluation of five data-driven feature selection methods. RF was used on Set 1 to evaluate the performance.

Feature selection method	Number of features	AUC	Sensitivity	Specificity
Recursive feature elimination	120	0.885	0.730	0.868
Relief	99	0.885	0.729	0.870
Information gain	119	0.884	0.725	0.871
glmStepAIC	57	0.874	0.714	0.872
Selection-by-filter	66	0.871	0.713	0.864

AUC: Area Under Curve

Table S2. Classification performance of nine ML algorithms in predicting postpartum depression on Set 1.

Model	AUC	Sensitivity	Specificity	Accuracy
RF	0.881	0.726	0.868	0.789
SVM	0.868	0.797	0.789	0.793
GBM	0.858	0.689	0.870	0.779
AdaBoost	0.856	0.717	0.837	0.777
RPART	0.785	0.648	0.810	0.726
NB	0.783	0.59	0.831	0.671
kNN	0.768	0.928	0.432	0.632
LR	0.703	0.630	0.676	0.653
NNET	0.697	0.660	0.633	0.646

AUC: Area Under Curve; RF: Random Forest; GBM: Stochastic Gradient Boosting; SVM: Support Vector Machines; RPART: Recursive Partitioning and Regression Trees; NB: Naïve Bayes; kNN: k-Nearest Neighbor; LR: Logistic Regression; NNET: Neural Network.

Table S3. **Classification performance of nine ML algorithms in predicting postpartum depression on Set 2.**

Model	AUC	Sensitivity	Specificity	Accuracy
RF	0.889	0.745	0.862	0.797
SVM	0.868	0.791	0.793	0.792
GBM	0.863	0.708	0.864	0.785
AdaBoost	0.861	0.733	0.831	0.782
NB	0.805	0.588	0.852	0.683
RPART	0.795	0.665	0.806	0.734
kNN	0.787	0.928	0.47	0.648
LR	0.716	0.627	0.695	0.661
NNET	0.712	0.648	0.674	0.656

AUC: Area Under Curve; RF: Random Forest; GBM: Stochastic Gradient Boosting; SVM: Support Vector Machines; RPART: Recursive Partitioning and Regression Trees; NB: Naïve Bayes; kNN: k-Nearest Neighbor; LR: Logistic Regression; NNET: Neural Network.

Table S4. **Classification performance of nine ML algorithms in predicting postpartum depression on Set 3.**

Model	AUC	Sensitivity	Specificity	Accuracy

RF	0.881	0.726	0.865	0.788
SVM	0.857	0.785	0.782	0.783
GBM	0.855	0.689	0.87	0.779
AdaBoost	0.854	0.717	0.837	0.776
NB	0.792	0.557	0.875	0.671
RPART	0.786	0.661	0.806	0.732
kNN	0.774	0.92	0.462	0.642
LR	0.703	0.628	0.677	0.652
NNET	0.703	0.642	0.674	0.647

AUC: Area Under Curve; RF: Random Forest; GBM: Stochastic Gradient Boosting; SVM: Support Vector Machines; RPART: Recursive Partitioning and Regression Trees; NB: Naïve Bayes; kNN: k-Nearest Neighbor; LR: Logistic Regression; NNET: Neural Network.

Table S5: **Top 20 contributing features in the four best performing models**. Collectively, there were 50 features returned by the top 4 models, ranked by frequency of occurrence in the top 20 features for each model. 'RF rank' for BF5WEEKS, for instance, means that the feature was ranked at the 1st in the RF model but ranked at the 3rd in the GBM model. The "Description" column refers to what each feature means or the survey question.

Features	Frequency	RF rank	GBM rank	AdaBoost rank	SVM rank	Description from PRAMS
BF5WEEKS	4	2	2	5	3	Number of weeks spent breastfeeding the baby
BPG_DEPRS	4	3	3	4	7	Depression before pregnancy
MAT_AGE_NAPHSIS	4	9	5	13	9	Maternal age grouped
STRS_T_G	4	1	1	1	1	Total number of stresses during the 12 months before childbirth grouped
INCOME7	3	6	15	NA	2	Total household income during the 12 months before childbirth
MAT_ED	3	7	8	NA	4	Maternal education
PRE_DEPR	3	NA	12	19	16	Pre-pregnancy check for depression/ anxiety
PREG_TRY	3	NA	6	6	17	Trying to get pregnant
STRS_BIL	3	12	7	NA	5	Stress -- couldn't pay rent, mortgage, or other bills
LTH_HOSP	2	NA	16	7	NA	Hospital baby stay -- length of time
MAT_RACE	2	NA	4	11	NA	Maternal race/ethnicity
MM_NOMD	2	NA	19	17	NA	No medical risk factors?
PNC_VST_NAPHSIS	2	NA	18	14	NA	Number of prenatal care visits grouped
PRE_DDS	2	NA	20	20	NA	Pre-pregnancy teeth cleaned
SEX	2	NA	9	2	NA	Gender of infant
VITAMIN	2	NA	13	NA	20	Multivitamin -- Number of times taking a

Features	Frequency	RF rank	GBM rank	AdaBoost rank	SVM rank	Description from PRAMS
						multivitamin, a prenatal vitamin, or a folic acid vitamin/week
WIC_PREG	2	NA	10	NA	8	WIC participation during pregnancy
BC_GEST	1	5	NA	NA	NA	Gestational age (Days)
DDS_TALK	1	NA	17	NA	NA	A dental or other health care worker talked about how to care for one's teeth and gums
FLUPREG	1	NA	NA	10	NA	Flu vaccination during pregnancy
GRAM_NAPHSIS	1	15	NA	NA	NA	Birth weight grouped into 250-gram intervals
HI_MEDIC	1	NA	NA	NA	15	Insurance paid by -- Medicaid
HI_WORK	1	NA	NA	NA	11	Insurance paid by -- job
IDOB_MTH	1	17	NA	NA	NA	Infant birth -- month
INC_NDEP	1	NA	NA	18	NA	Income -- dependents (+self)
INFQ_AGE_MOD	1	4	NA	NA	NA	Infant age (days) (cleaned version)
INSWORK	1	NA	NA	NA	10	Insurance paid by -- job
KOTELCHUCK	1	NA	NA	8	NA	Kotelchuck Index
MM_LMP	1	19	NA	NA	NA	Month of last menstrual period
MODECONT	1	NA	11	NA	NA	Mode of Contact
MOM_BMI	1	13	NA	NA	NA	Maternal body mass index
MOM_CM	1	16	NA	NA	NA	Maternal height -- centimeters
MOM_INCH	1	20	NA	NA	NA	Moms height -- inches
MOM_WT	1	10	NA	NA	NA	Maternal weight before pregnancy
MOMLBS	1	8	NA	NA	NA	Maternal weight gain
OTH_TERM	1	NA	NA	9	NA	Pregnancy history – Other terminations?
PGWT_GN_DK	1	11	NA	NA	NA	Pregnancy weight gain/lost
PNC_1ST	1	14	NA	NA	NA	First prenatal care visit -- number
PNC_MTH	1	NA	NA	12	NA	Month of first prenatal care visit
PP_MEDIC	1	NA	NA	NA	6	Insurance paid by -- Medicaid
PP_WORK	1	NA	NA	NA	13	Insurance paid by -- job

Features	Frequency	RF rank	GBM rank	AdaBoost rank	SVM rank	Description from PRAMS
PRE_DIET	1	NA	NA	16	NA	Pre-pregnancy dieting
PRE_EXER	1	NA	NA	3	NA	Pre-pregnancy exercise 3+ days/week
SMK6C_NW	1	NA	NA	NA	19	Changing smoking last 3 months of pregnancy and now
SMK6C_PG	1	NA	NA	NA	14	Changing smoking during pregnancy
SMK6C_PP	1	NA	NA	NA	12	Changing smoking 3 months before pregnancy and now
STRS_DRG	1	NA	NA	NA	18	Stress -- someone close had a problem with drinking or drugs
TLK_DPRS	1	NA	14	NA	NA	Health care worker talk about depression
TLK_HIVT	1	NA	NA	15	NA	Health care worker talk about testing for HIV
TOD_MTH	1	18	NA	NA	NA	Today -- month

NA: Not Available; RF: Random Forest; GBM: Stochastic Gradient Boosting; SVM: Support Vector Machines

Table S6. Survey question: Infant living at the time of the PRAMS report.

	Postpartum depressed	Healthy
Yes	7,470	48,817
No	220	197

p-value < 2.2e-16; odds ratio: 7.30; Relative Risk: 3.96
